# A Prickly Problem: Genome Skimming Reveals Varying Levels of Phylogenetic Diversity in the Freshwater Crayfish *Euastacus armatus* Complex (Parastacidae) With Implications for Taxonomy and Conservation

**DOI:** 10.1002/ece3.73428

**Published:** 2026-04-20

**Authors:** Christopher M. Austin, Shane T. Ahyong, Adam Miller, Tarmo A. Raadik, Mark Lintermans, Rob McCormack, Sylvia Zukowski, Michael P. Hammer, Frederic Grandjean, Dean M. Gilligan, Matt Beitzel, Nick S. Whiterod

**Affiliations:** ^1^ School of Life and Environmental Sciences Deakin University Geelong Victoria Australia; ^2^ Research Institute for the Environment and Livelihoods Charles Darwin University Darwin Northern Territory Australia; ^3^ Museum and Art Gallery of the Northern Territory Darwin Northern Territory Australia; ^4^ Australian Museum Research Institute Australian Museum Sydney New South Wales Australia; ^5^ School of Biological, Earth & Environmental Sciences UNSW Kensington New South Wales Australia; ^6^ Cesar Australia Brunswick Victoria Australia; ^7^ Department of Energy, Arthur Rylah Institute for Environmental Research Environment and Climate Action Heidelberg Victoria Australia; ^8^ Centre for Applied Water Science University of Canberra Canberra Australian Capital Territory Australia; ^9^ Fish Fondler Pty Ltd Bungendore New South Wales Australia; ^10^ Australian Aquatic Biological Pty Ltd Karuah New South Wales Australia; ^11^ Nature Glenelg Trust Victor Harbor South Australia Australia; ^12^ Laboratoire Ecologie, Biologie des Interactions, Équipe Ecologie, Evolution, Symbiose Université de Poitiers Poitiers France; ^13^ Bush Heritage Australia Surfside New South Wales Australia; ^14^ City and Environment Directorate, ACT Government Dickson Australian Capital Territory Australia; ^15^ Goyder Institute for Water Research CLLMM Research Centre Goolwa South Australia Australia; ^16^ School of Biological Sciences Adelaide University Adelaide South Australia Australia

**Keywords:** *Euastacus bispinosus*, *Euastacus yarraensis*, genome skimming, outlying populations, Parastacidae, phylogenetics, relictual, spiny crayfish

## Abstract

Accurate resolution of species taxonomies and natural distributions is paramount to enable effective conservation and management. But this can be a challenge in systems that lack sufficient genomic resources and systematic research, and when species ranges are dynamic due to population decline, human‐mediated introduction, or responses to climate change. This is particularly true for many of the world's 690 species of freshwater crayfish, including Australia's endemic spiny crayfish from the *Euastacus* genus. In this study, we use genome skimming to successfully recover whole mitogenomes and nuclear markers to examine the molecular taxonomy, phylogeography and range uncertainty in a wide‐ranging complex, which includes the nationally Vulnerable Murray crayfish *Euastacus armatus*, the nationally Endangered Glenelg spiny crayfish *E. bispinosus* and the Southern Victorian spiny crayfish *E. yarraensis*. The analyses revealed high levels of genetic structure in both *E. armatus* and *E. yarraensis*, including sufficiently divergent lineages within *E. yarraensis* that potentially warrants independent taxonomic recognition. Furthermore, analyses resolved the status of *E. armatus* populations persisting outside known contemporary ranges as being relictual and of high conservation importance. Whilst one outlying population of *E. bispinosus* was confirmed as relictual, another was revealed as being translocated. This study demonstrates genome skimming as a powerful technique for generating molecular data to support key elements of conservation for freshwater crayfish and other species.

## Abstract 2

Whole mitogenome and nuclear molecular analyses clarify conservation uncertainty in wide‐ranging freshwater crayfish complex. We reveal high levels of genetic structure and resolves the conservation status of several outlying populations, and divergent lineages that potentially warrants independent taxonomic recognition. This study demonstrates genome skimming as a powerful technique for generating molecular data to support key elements of conservation for freshwater crayfish and other species.

## 1 Introduction

To avert the biodiversity crisis the world is currently experiencing, effective conservation prioritisation, planning and management is required to prevent the loss of species, ecosystems and genetic diversity (Dobrzycka‐Krahel et al. 2024; Hunter et al. 2021; Tickner et al. 2020). A multitude of correlative and quantitative tools are now available for addressing critical knowledge gaps to support conservation decision‐making. In particular, molecular methods are used widely for quantification of genetic variation within and between species, providing critical insights into species taxonomies, demographic histories and evolutionary trajectories (Allendorf et al. 2022; Hoelzel 2023). The uptake of genomic data and novel analytical tools has increased rapidly over the last decade due to advances in next‐generation DNA sequencing technologies. However, progress in this field relies on evaluating the efficacy of emerging methods for addressing key elements of conservation biology and to reliably guide conservation (Allendorf et al. 2022; Hoelzel 2023; Kardos et al. 2021; Onley et al. 2021; Willi et al. 2022).

Testing taxonomic boundaries developed using traditional methods, particularly to uncover undiagnosed cryptic species (Adams et al. 2014), identifying genetic and phenotypic variation among conspecific populations, and determining species' natural geographic ranges is fundamental to the conservation process (Lawton 1993). Yet a challenge for mapping geographic ranges is that they can change as a result of both natural and anthropogenic influences (e.g., climate and environmental change and translocations). This can have significant influence on species ecologies, vicariant divergence through time, selective pressure in novel habitats, and thus have elevated conservation importance (Allendorf et al. 2022; Gaston 2003). Geographically outlying populations are of special relevance as they may represent remnants of a historically more substantial distribution, carry novel alleles, and have elevated conservation importance (Bradbury et al. 2019; Hammer et al. 2010; Owen and Taylor 2023). Alternatively, disjunct populations originating from human‐mediated translocations can be of lesser conservation importance (Lundgren et al. 2024).

The taxonomic status and natural geographic ranges of many of the world's 690 freshwater crayfish species remain poorly resolved (Crandall and De Grave 2017; Guiaşu and Labib 2021). Human‐mediated introduction of freshwater crayfish is a significant problem worldwide, complicating the understanding of historical distributions whilst threatening native species at both local and global scales (Austin and Ryan 2002; Horwitz 1990; Lodge et al. 2012; Soto et al. 2023). Additionally, one‐third of all species are considered at risk of extinction with many experiencing substantial range contractions over the last century (Richman et al. 2015; Sayer et al. 2025), while others have shifted range in response to changes in climate and habitat suitability (Dobrzycka‐Krahel et al. 2017; Hossain et al. 2018; Thomas 2010). These factors, coupled with high levels of morphological conservatism among closely related species, can confound the ability to accurately delimit species and their contemporary distributions and therefore hampers effective conservation (Hending 2025).

The above issues continue to hinder the conservation of Australia's particularly species rich, endemic freshwater crayfish fauna, including those belonging to the highly threatened genus *Euastacus*. Many species of *Euastacus* are large and charismatic and are commonly known as spiny crayfish or ‘prick(ly) backs’. They typically have conspicuous spines, are slow growing, and are mostly adapted to cooler pristine flowing aquatic systems (Crandall and De Grave 2017; Furse and Coughran 2011b; Richman et al. 2015). Species of *Euastacus* face a range of threats relating to habitat degradation, flow alteration, and harvest pressure (Furse and Coughran 2011a). The larger species of the genus have been subject to translocations outside their natural ranges (Horwitz 1990).

The nationally Vulnerable Murray crayfish *Euastacus armatus* (Von Martens, 1866) is an iconic species being the largest and most widely distributed member in the genus and second largest freshwater crayfish in the world (Gilligan et al. 2007; Morgan 1986, 1997). Although there is generally little dispute over the taxonomic validity of the species (Morgan 1986; Riek 1969; Shull et al. 2005), uncertainty exists about some populations, both historically (Clark 1941) and more recently (Street et al. 2010; Versteegen and Lawler 1996). The status of two outlying and isolated northern populations of *E. armatus* , each separated from the core distribution of the species, and from each other, by hundreds of kilometres of contemporary river distance, is unclear (Gilligan et al. 2007; Morgan 1986).

Limited genetic data suggests that *E. armatus* forms a complex of sibling species with two other large‐bodied species with allopatric distributions: the nationally Endangered Glenelg spiny crayfish *Euastacus bispinosus* (Clark 1941), and the Southern Victorian spiny crayfish *Euastacus yarraensis* (McCoy, 1888) (Avery and Austin 1997; Shull et al. 2005). At present, genomic resources for species of the *Euastacus armatus* complex are poorly represented in international databases (see Figure S1) compared with other widespread species and speciose genera of crayfish (Gan et al. 2018; Grandjean et al. 2017; Hammer et al. 2024; Nguyen et al. 2005; Tan et al. 2021). Consequently, broad‐based genomic analyses, coupled with comprehensive spatial sampling, are warranted given the heightened conservation significance of this important group of crayfish.

Genome skimming is a relatively new molecular genetic approach which uses shallow shotgun sequencing to recover organelle genomes and high copy number nuclear loci at relatively low cost that has been used mostly in phylogenetic studies (Taite et al. 2023; Kou and Poore 2025). A recent study on northern Australian freshwater crayfish (Hammer et al. 2024) demonstrated that in addition to recovering full mitogenomes, this approach can recover the full ribosomal contig (18S, ITS1, 5.8S, ITS2, 28S loci) and the full histone contig (H2A, H2B, H3 and H4 and intergenic regions) in crayfish. This study also demonstrated that this approach can be used to provide insights into species boundaries and patterns of intra‐species variation (phylogeography).

The aim of this study is to explore the molecular taxonomy and phylogeography of the *E. armatus* complex to help overcome current knowledge gaps impacting the conservation of this group of species. This is achieved through genome skimming to generate complete mitogenomes and nuclear DNA sequences for *E. armatus* , *E. bispinosus* and *E. yarraensis* collected from across their contemporary ranges with the resulting data used for comprehensive molecular taxonomic and phylogeographic analyses. This comprehensive data set sheds new light on the taxonomy, demographic and evolutionary history of this complex, and provides an essential resource for management. Outputs from this study emphasise the power of genome skimming for generating essential genomic resources to support conservation decision‐making in crayfish and other threatened species.

## 2 Methods

### 2.1 Study Species and Sample Collection

**FIGURE 1:**
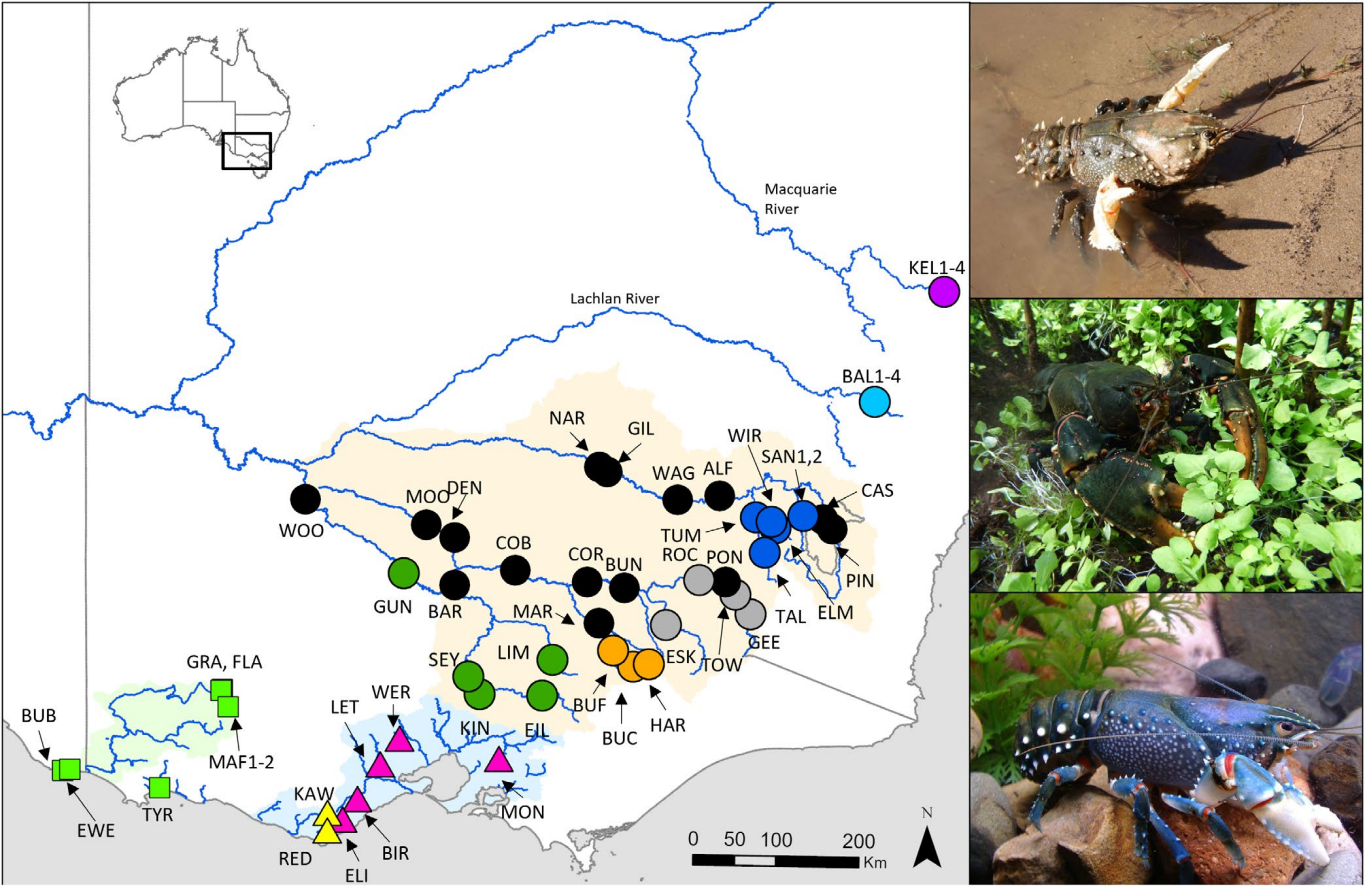
Map of south‐eastern Australia showing sample collection sites (coloured circles with site code) used in this study with contemporary distribution for *E. armatus* (top right image: Nick Whiterod) in light brown shading, *E. bispinosus* (middle right image: David Mossop) in light green shading and *E. yarraensis* (lower right image: Rob McCormack) in light blue shading. Refer to Table S1 for detailed description of sampling sites and georeferencing and location description for each site codes. Further details of sample colour coding are provided in the text.

The three members of the *E. armatus* complex occur allopatrically throughout waterways of southeastern Australia (Figure 1). *Euastacus armatus* inhabits the Murray and Murrumbidgee rivers basins and tributaries but has a discontinuous distribution that includes two outlying northern populations recorded from the upper reaches of the Abercrombie River (Lachlan River Basin) and the Cudgegong River (Macquarie‐Bogan Rivers Basin) (Gilligan et al. 2007; Morgan 1997). *Euastacus bispinosus* occurs across the southerly flowing Glenelg River Basin with populations in the lower reaches and in headwater streams in the Grampians National Park (Morgan 1986). Outlying populations persist in karst‐rising springs in the Millicent Coast Basin to the west (Miller et al. 2014; Whiterod et al. 2015) and in the Fitzroy River and tributaries (Portland Coast Basin) to the east, with the latter considered likely to be from translocations (Miller et al. 2018). *Euastacus yarraensis* is found from the Bunyip and Yarra River basins in the east extending south‐westerly to the Otway Coast Basin in southerly draining river basins (Morgan 1986).

Our study utilised tissue samples obtained broadly across the range of *E. armatus* , *E. bispinosus* and *E. yarraensis* (Table S1). These tissues samples were substantially collected for previous studies (Avery and Austin 1997; Coughran et al. 2015; Miller et al. 2014; Whiterod et al. 2017), from specimens maintained in private or museum collections and from a small number of new wild collected specimens (Table S1). With few exceptions, a single individual was sampled per site as crayfish generally show very low levels of intra‐population variation (Austin et al. 2022; Nguyen et al. 2005; Shull et al. 2005). Two exceptions were the outlying populations of *E. armatus* in the Abercrombie River (*n* = 4) and Cudgegong River (*n* = 4) as preliminary data indicated unexpected divergence levels in crayfish from these locations. The giant spiny crayfish, *Euastacus spinifer* (Heller, 1865), is the most closely related species to the *E. armatus* complex (Shull et al. 2005), and samples from Hastings and Manning river basins (*n* = 3) from northern coastal New South Wales (locations not shown) were included as an outgroup and for comparative purposes.

### 2.2 Genome Skimming

#### 2.2.1 DNA Sequencing

DNA extraction, quality control and library preparation are as described in related studies (Grandjean et al. 2016; Hammer et al. 2024; Tan et al. 2019, 2021). The genome skimming methods followed those outlined in Gan et al. (2014) for mitogenome recovery and more recently extended to the recovery of 18S and 28S ribosomal genes by Grandjean et al. (2017) and Tan et al. (2021), and the full 18S‐28S gene cluster by Hammer et al. (2024). Samples were sequenced on an Illumina NovaSeq instrument at the Deakin Genomics Centre (Geelong, Australia) or the Ramaciotti Centre for Genomics (Sydney, Australia) using either 2 × 150 bp or 2 × 250 bp paired end reads with the aim to achieve a minimum of 1.0 G bases of raw reads for each sample.

### 2.3 Bioinformatics

Mitogenomes were assembled essentially using the methods outlined by Grandjean et al. (2017) with the exception that reads were trimmed using BBduk and de novo assemblies were determined using the SPAdes software (either implemented under the Geneious Prime software or using the command line version obtained from the GitHub software repository) (https://github.com/ablab/spades).

For most samples, ~3–5 million reads were sub‐sampled where the data allowed for the *de novo* assemblies with the contigs mapped against existing circularised mitogenomes from the same or a closely related species. The 18S and 28S genes and associated genetic elements were initially identified from the assembled contigs by mapping to baits using *Euastacus* 18S and 28S sequences. Baits were obtained from the National Center for Biotechnology Information (NCBI) nucleotide database and then validated; sequenced obtained from the first samples assembled were used as references for the subsequent mapping of assemblies. For the majority of samples, the mitogenome was obtained as a single contig, and frequently the full 18S–28S gene cluster was recovered as a single or as 2–3 overlapping contigs. Recovery of the full ribosomal contig for some *E. armatus* samples was more problematic as they were found to have a particularly long ITS1 region containing a long‐duplicated element (~500 bp). When contigs did not fully encompass the target genes, iterative mapping was used to extend the contigs until they overlapped (see Tan et al. 2019) or the assembly was re‐run using additional reads when available. While the full 18S–28S contig was recovered from all but one sample, the ITS1 was not included in the analyses due to its high length variability among species. Recovery of histone genes was also achieved (Hammer et al. 2024; Tan et al. 2021), but as these showed minimal divergence among these *Euastacus* species, these were also excluded from the study.

Once the target gene regions were recovered from the SPAdes assemblies, the full set of trimmed sequence reads were mapped using the Geneious Primer reference mapper, set at either 5 or 10% maximum mismatch per read, to test the integrity of the assembled contigs and generate coverage statistics. Reads from the NCBI SRA were downloaded for the three published *Euastacus* mitogenomes ( *E. armatus* , *E. spinifer* and *E. yarraensis* ) to re‐check the mitogenome assemblies and to extract the full set of ribosomal genes and genetic elements.

Annotation of all mitogenomes was undertaken by using GenBank accessions for *E. armatus* , *E. yarraensis* and *E. spinifer* . Where minor differences in annotations were apparent among the three species, the consensus was chosen. The ribosomal genes and spacers were annotated based on NCBI nucleotide accessions for crayfish. The annotation of the 18S and the 5.8S genes was straightforward but the 28S gene is more problematic for crayfish and for decapod crustaceans more generally. For this study annotation for this gene was taken from the studies of Grandjean et al. (2017) and Tan et al. (2021) while acknowledging that alternative annotation pipelines may yield slightly different gene boundaries. The sequences intervening the 5.8S 3′ boundary and the 28S 5′ boundary are referred to as Internal Transcribed Spacer 2 (ITS2) ribosomal element and the sequences downstream of the 28S 3′ prime boundary as the partial External Transcribed Spacer (ETS).

### 2.4 Tree and Network‐Based Analyses

The sequence data were aligned using MAFFT (Version: 1.5.0) with default settings. Phylogenetic trees were estimated using IQ‐TREE essentially as described by Gan et al. (2018) using the IQ‐TREE web server (Trifinopoulos et al. 2016). Individual trees were estimated for each of the 2 major sets of genetic data (mitochondrial and concatenated 18S, 5.8S, ITS2, 28S and ETS) and the data were also concatenated to generate a total evidence tree. The data were partitioned on the basis of the major genes/spacers. For the mitogenome data set, each of the protein coding genes and the 12S and 16S genes were extracted and concatenated, making 15 partitions. The ribosomal genes and spacers were partitioned into 18S, 5.8S, ITS2, 28S and ETS.

In addition, to obtain a total evidence maximum‐likelihood tree from the complete mitochondrial sequences (inclusive of the tRNA and control region (CR) sequences), they were analysed without partitioning. For these analyses a 27 bp block of the CR sequences was deleted as it was dominated by a dinucleotide repeat and was hypervariable within and between species. Next, network‐based analyses were conducted for each species using the Median Joining Network procedure implemented using PopART (Leigh et al. 2015) based on the same data.

To examine the taxonomic utility and divergence levels among the ribosomal genes and spacer regions, genetic distances were calculated using the Jukes‐Cantor method (Jukes and Cantor 1969) and relationships depicted using UPGMA. For the complete ribosomal data set distances were calculated using pairwise deletion and with gaps included using P*R*O*P (www.rs.tus.ac.jp/bioinformatics/prop, Nishimaki and Sato 2021). All trees were visualised and edited using the Geneious Prime interface.

### 2.5 Species Delimitation Analysis

A tree‐based species boundary analysis was performed firstly using the multi‐rate Poisson Tree Processes (bPTP) method (Zhang et al. 2013). The bPTP software and webserver (https://species.h‐its.org) operates upon pre‐existing trees using a Bayesian approach, giving probabilities for clusters conforming to species hypotheses in rank order. Trees estimated as previously described were used as input in Newick format. A total of 200,000 generations were used with a burn in of 0.1.

We also used the ASAP web server which implements the ‘Assemble Species by Automatic Partitioning’ pipeline (Puillandre et al. 2012, 2021) to infer species delimitation based on the pair‐wise Juke‐Cantor distance using the aligned FASTA files for the various data sets. Number of steps was set at 10 and the best outcome in terms of species boundaries was determined by the lowest ASAP score and associated probability.

## 3 Results

### 3.1 Genome Skimming Data

Mitochondrial and nuclear DNA data sets were successfully generated for 55 individuals representing *E. armatus* (40), *E. bispinosus* (7), *E. yarraensis* (6) and *E. spinifer* (2). Raw sequences generated by this study are available on the NCBI SRA under BioProject PRJNA1152318 and project ‘DKN.TSI Euastacus’, from the Threatened Species Initiative database accessed via Bioplatforms Australia Data Platforms (https://data.bioplatforms.com) (Table S2). Existing raw sequence data (Gan et al. 2016, 2018) from three *Euastacus* individuals representing *E. armatus* , *E. yarraensis* and *E. spinifer* were downloaded from the NCB SRA and also included in the dataset (see details in Tables S1 and S2).

Across all samples an average of 2.42 Gb of trimmed raw sequence data were obtained or available (range: 0.24–4.96 gb) from which the full mitochondrial genome and ribosomal sequences were extracted (Table S2). Complete mitochondrial genomes were recovered from each sample with an average coverage of 387× (range: 17 to 12,459×; Table S2), and the mitogenome lengths ranged from 16,357 to 16,388 bp. The three samples analysed in previous mitogenome studies (Gan et al. 2016, 2018) were each found to have longer CR sequences than were found from the original assemblies, with the newer assemblies being consistent with the newly sequenced samples of the same species for this study.

The 18S gene was recovered from all samples and was 1884 bp in length for *E. armatus* , *E. bispinosus* and *E. yarraensis* and 1883 bp for *E. spinifer* with an average coverage of 1219× (range: 24 to 4049×). A ‘5.8S‐28S’ contig was also extracted, including, in sequence, the 5.8S gene, ITS2 region, the 28S gene and the partial ETS region. These elements were recovered for an equivalent alignment length (range: 6790–7248 bp) for all samples, with the exception of sample MON ( *E. yarraensis* ) for which only a partial 28S gene could be recovered. Average coverage for the 5.8S–28S contig was 981× (range: 21 to 2375×; see Table S2 for more details).

### 3.2 Phylogenetic, Network and Taxonomic Analyses

**FIGURE 2:**
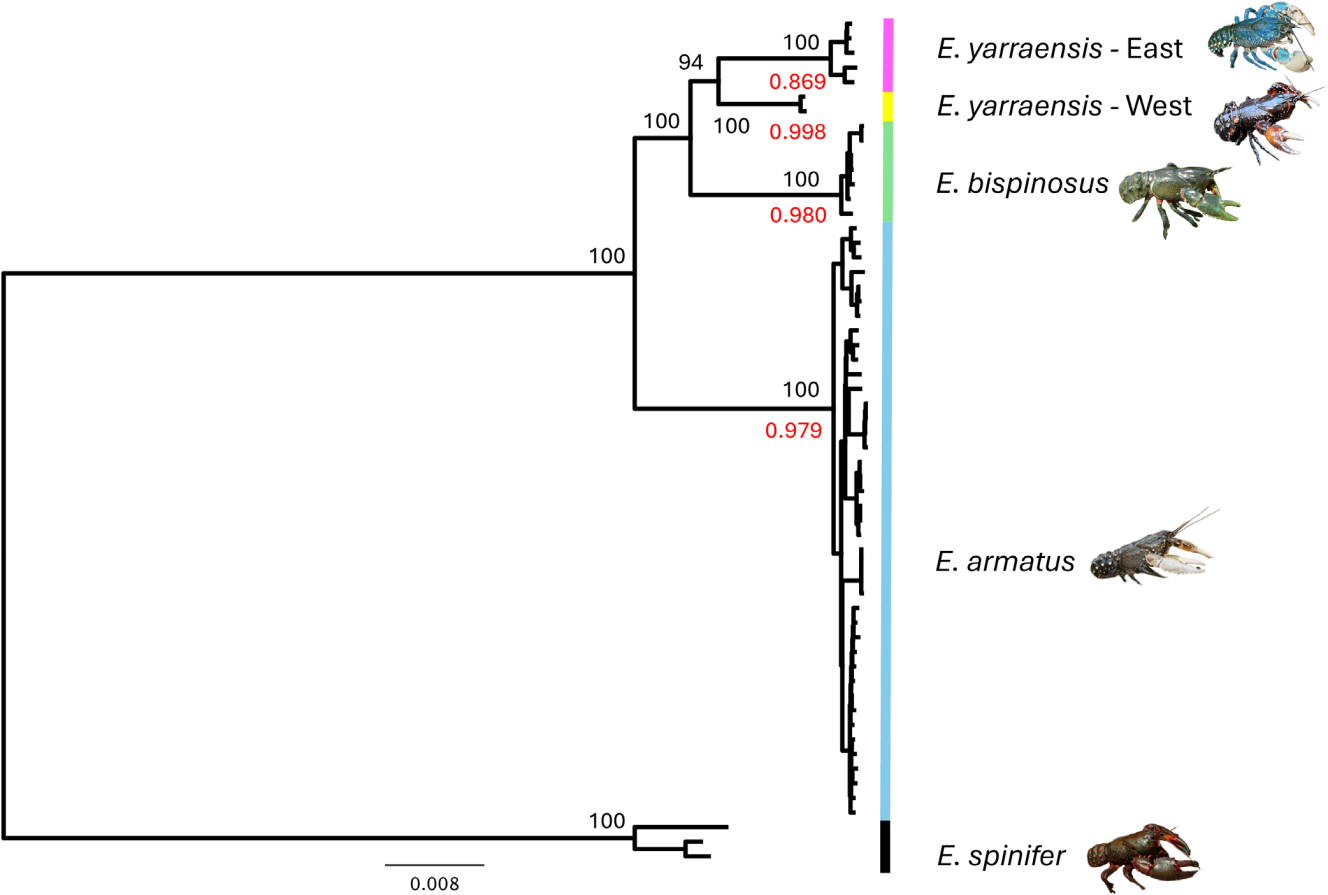
Total evidence maximum likelihood tree calculated for 4 species of *Euastacus* using IQ‐TREE with 20 data partitions (13 mitochondrial protein coding genes and 2 rRNA genes; 18S, 5.8S and 28S genes and ITS2 and ETS sequences), comprising an alignment of 22,289 bp. Bootstrap support given in black font for the deep nodes and the highest Bayesian probabilities from the bPTP species delimitation tool in red font.

A maximum likelihood phylogenetic reconstruction based on the combined mitochondrial and ribosomal data (a total of 20 data partitions) revealed four well supported (94%–100%) monophyletic clades (excluding the outgroup) (Figure 2). All *E. armatus* and *E. bispinosus* samples were assigned to monophyletic clades in accordance with accepted taxonomies. In contrast, samples of *E. yarraensis* formed two distinctive and well supported clades (100%), with samples (RED, Aire River; KAW, Loves Creek, Gellibrand River) in the western part of the species' range, being genetically distinct from those the samples in the Barwon River Basin (ELI and BIR) and further eastward. Divergence among samples within clades was generally low relative to between‐clade divergence (Figure 2).

**FIGURE 3:**
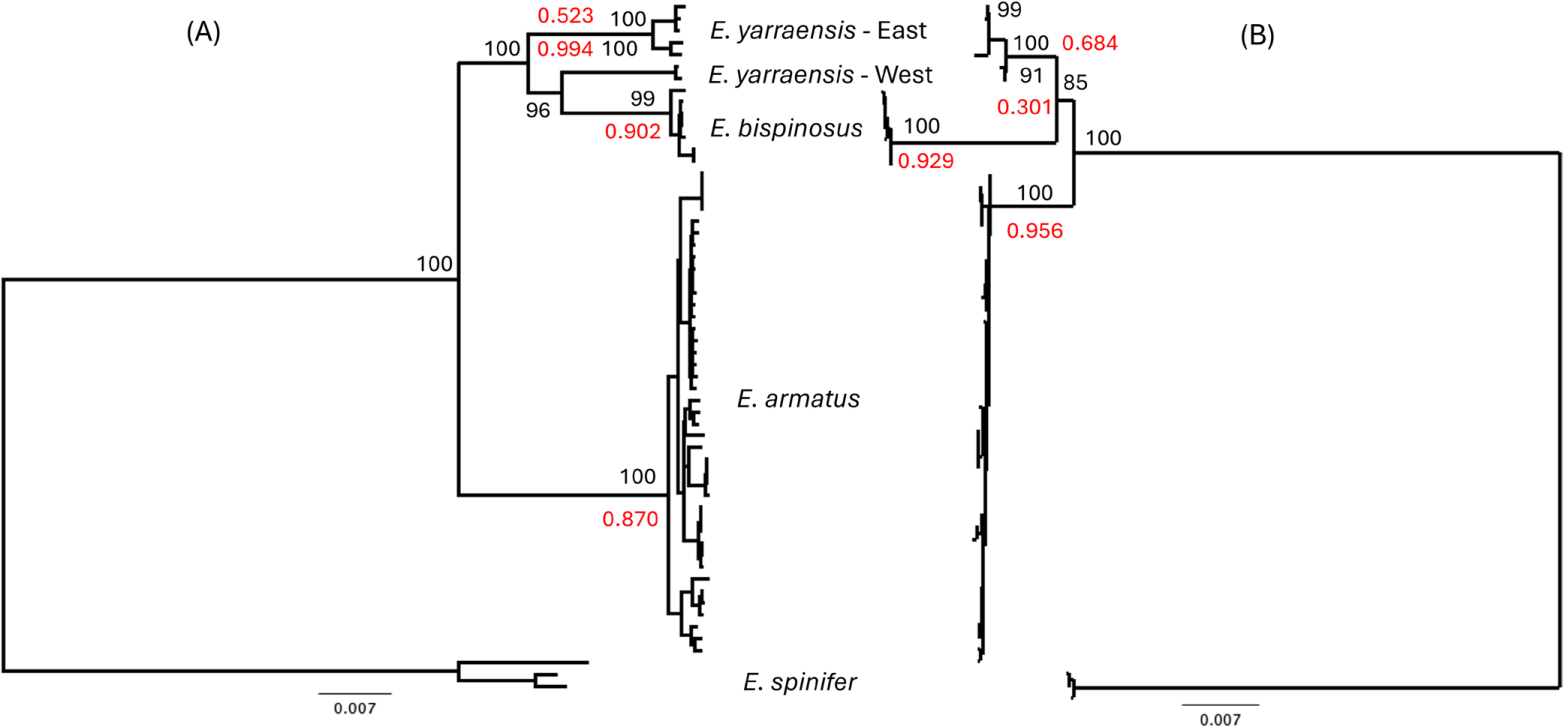
Maximum likelihood trees calculated separately for the (A) mitochondrial‐based genes and (B) ribosomal cluster elements. Bootstrap support given in black font for the deep nodes and the highest Bayesian probabilities from the bPTP species delimitation tool in red font.

Separate phylogenetic reconstructions performed on the mitochondrial and ribosomal DNA datasets provided broadly congruent tree topologies (Figure 3) except for the two *E. yarraensis* clades. The mitochondrial DNA reconstruction indicated the RED and KAW samples to be again differentiated from the *E. yarraensis* samples from the eastern portion of the species distribution, both forming highly supported (100%) monophyletic clades. However, in this analysis the RED and KAW samples formed a well‐supported (96%) sister relationship with the *E. bispinous* clade, albeit in a basal position. In contrast, the ribosomal DNA reconstruction found all *E. yarraensis* samples to form a well‐supported (100%) monophyletic clade, albeit with the eastern and western samples being distinct, forming well‐supported (99% and 91%) subclades, and showing greater divergence than was evident within the other two species.

Multidimensional scaling (MDS) analysis (Figure S2A) of taxonomic relationships using the pairwise genetic distance values from the full mitochondrial data set separates *E. armatus* and *E. spinifer* samples on axis 1 with positive scores from the *E. yarraensis* and *E. bispinosus* samples with negative scores. The *E. spinifer* samples are additionally separated from all samples on axis 2 with high negative scores. The *E. bispinosus* , western *E. yarraensis* , and eastern *E. yarraensis* samples are the most similar, but each set of samples cluster distinctly on the basis of variation on the combined axes. The differentiation between the two groups of *E. yarraensis* samples is greatest on axis 2, with the *E. bispinosus* samples placed in an intermediate position.

The outcomes from the species delimitation pipelines were largely congruent with each other and support the above interpretations from the phylogenetic trees. For the combined 20 partition data set, ASAP identified four partitions (*p* = 0.204, Threshold Distance (TD) = 0.0131), which correspond to the groupings with the highest Bayesian probabilities indicated in red font (Figure 2). These pipelines gave similar results for the mitochondrial tree (Figure 3A). Four ASAP partitions were identified with a *p*‐value of 0.0099 and TD of 0.0193. While the 4 highest bPTP probabilities identified these same groups, they were generally reduced compared with the 20‐partition tree. For the ribosomal data, just three groups were identified (Figure 3B) corresponding to the current species concepts by ASAP (*p* = 0.00012; TD = 0.00376), which also received the highest bPTP values.

In terms of the overall evolutionary relationships among the three major clades, all analyses support *E. armatus* as the most distinct or divergent species, with the two more southerly distributed species, *E. bispinosus* and *E. yarraensis* representing sister clades.

#### 3.2.1 Phylogeography

**FIGURE 4:**
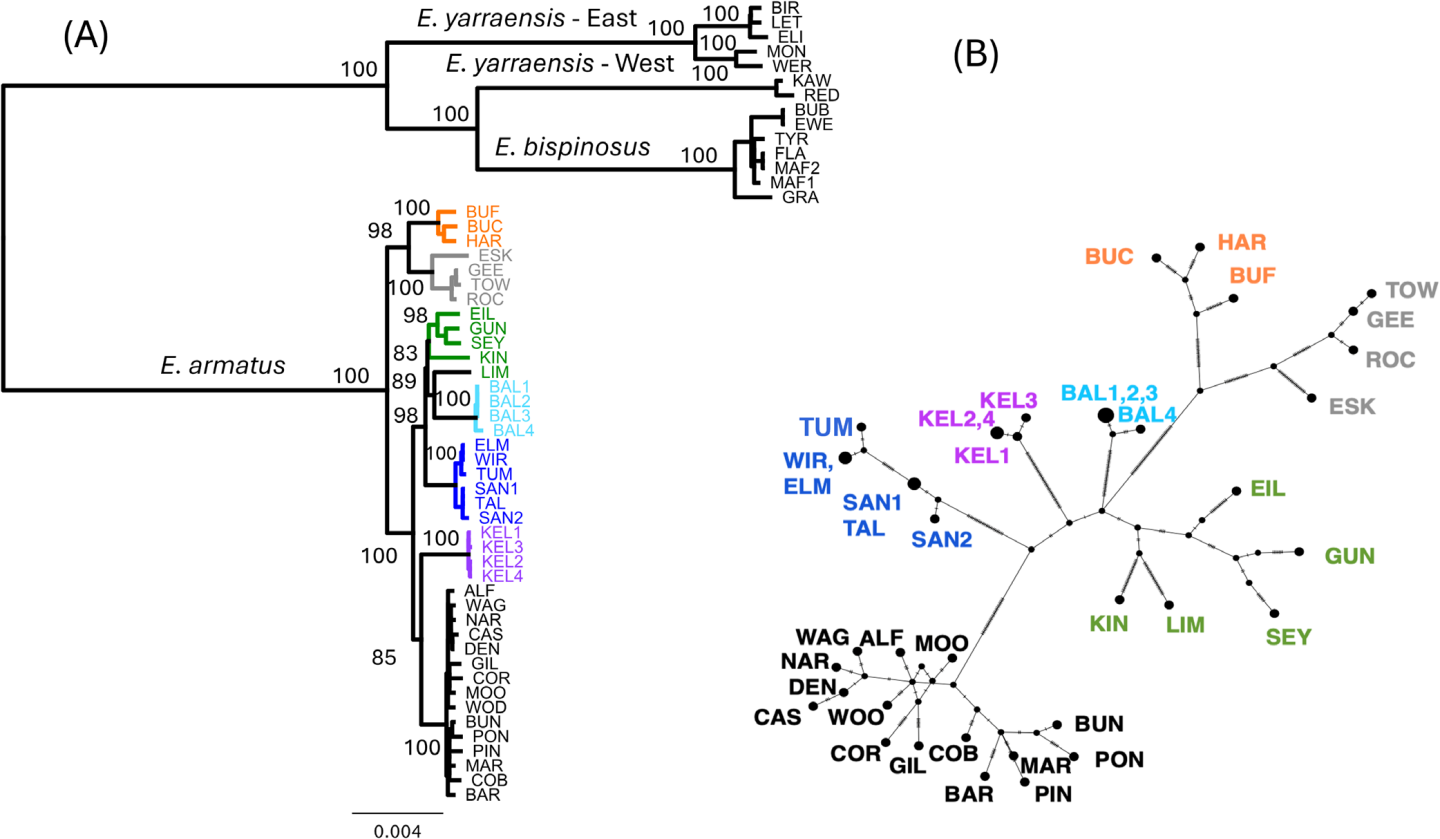
Representation of relationship among 41 samples of *E. armatus* using the full mitogenome data analysed using IQ TREE with the *E. bispinosus* and *E. yarraensis* samples as the outgroup (A); and a minimum spanning network for *E. armatus* samples (16,383 bp) (B). Distinct lineages or clusters of samples are colour coded and mapped on Figure 1.

Phylogeographic relationships among *E. armatus* samples were successfully estimated using the full mitochondrial data set (apart from the deletion of a 24 bp repetitive section from the CR) based on both phylogenetic (maximum likelihood) and network‐based analyses (Figure 4). Both analyses generated largely consistent results and indicate significant phylogeographic structuring within the species. Using the colour coding from Figure 1 the genetic patterns are dominated by strong geographic‐based differentiation between what is referred to as the lowland samples (‘black’ lineage), from almost exclusively the lower and larger reaches of the Murray‐Murrumbidgee river basins, and all other samples that themselves form a heterogenous group, but with restricted distributions. These latter samples are almost exclusively limited to the small creeks and rivers in headwater systems and include the Abercrombie and Cudgegong rivers, and tributaries of the Murray and Murrumbidgee rivers.

A total of seven geographically based clades can be identified (Figures 4 and S2B). The dominant ‘black’ clade shows relatively low genetic variation overall despite encompassing a significant geographic area ranging from the upper Murrumbidgee River (PIN) to the upper Murray River (PON), a river distance of approximately 2000 km (Figures 4 and S2B). Average similarity within the ‘black’ clade (99.92%) falls within the range seen for the other much more geographically restricted clade‐based groupings (average: 99.75%–99.99%) with a similar pattern also apparent from diversity analyses of the mitogenome dataset (Table S3). Almost all other samples fall into one of six distinct clades or clusters that are identified in both the phylogenetic and network analyses and are almost all associated with headwater river systems. Most noteworthy is that the four crayfish sequenced from each of the two northern outlying populations from Abercrombie River (BAL, light blue) and Cudgegong River (KEL, purple) (Figures 1 and 4) are genetically distinct from each other and from all other *E. armatus* samples.

Proceeding south, a fourth distinct clade (dark blue) is represented by six individuals from the adjacent Tumut and Goodradigbee rivers, tributaries of the upper Murrumbidgee River. These samples are genetically homogenous and quite distinct from samples of the widespread lineage (black) that were sampled nearby (ALF, PIN, CAS) from the upper Murrumbidgee River (Figure 1). The next geographically distinctive cluster of samples from the upper Murray River and its tributary, the Mitta Mitta River (grey clade), is also significantly divergent from the widespread black lineage with minor geographic overlap with the black clade (sample PON is located less than eight river kilometres upstream of the grey clade sample ROC) (Figure 1). The Ovens River, the next major tributary of the Murray River to the southwest, also has a cluster of three samples that have genetically divergent haplotypes (orange) located in its upper reaches. Despite the observation of samples of the black clade (COR, BUN, MAR) in the intervening river basins between the grey (Ovens River) and orange (upper Murray and Mitta Mitta rivers) lineages, each is divergent but shows a strong phylogenetic association forming a sister clade together distinct from all other *E. armatus* samples (98% support).

A seventh phylogeographic grouping is associated largely with the upper reaches of the southern drainages of the Goulburn River Basin, which joins the Murray River at Barmah. This group (dark green) is not as geographically and genetically coherent as the other distinct lineages identified above, with significant diversity among samples and with one member recorded from the mid‐Murray River at Gunbower National Park (GUN). Nevertheless, this group of samples is genetically distinct from the ‘black’ lineage, with which it is closest geographically and as a group is more closely related to the geographically discrete lineages from further north and east.

The MDS analysis using mitogenome‐based genetic distances among the *E. armatus* samples is consistent with the phylogenetic and network‐based analyses (Figure S2B). This analysis highlights the distinctiveness of the Murray‐Murrumbidgee clade (black circles) (negative scores on axis 1 and positive scores on axis 2) from the samples from the upper catchments (upper Murray and Mitta‐Mitta rivers (grey); upper Ovens River (orange); the Upper Murrumbidgee River tributaries (blue); the Cudgegong River (light blue), the Abercrombie River (purple), and the Goulburn River (dark green)). The latter group, largely from the Goulburn River, shows the highest level of within‐group variability.

The levels of intra‐specific divergence among samples of *E. bispinosus* from the phylogenetic analysis and network analysis (Figures 4 and S3A) are of a similar order of magnitude taking into account the species' more limited geographic range. The major geographic pattern of variation is the divergence of samples (EWE, BUB) from the outlying karst rising‐springs in the Millicent Coast Basin to the west, that are independent of the Glenelg River Basin. The other samples from the upper Glenelg and Wannon rivers, from higher elevation in the Grampians National Park, are relatively homogenous with the exception of a single sample from the upper Glenelg River (GRA) that is more divergent despite its geographical proximity to the other samples from this region. The sample from the outlying population in the Fitzroy River in the Portland Coast Basin (TYR), thought to be the result of historical translocation, clusters closely with the main group of *E. bispinosus* samples from the upper Glenelg and Wannon rivers.

As was apparent from the earlier analysis, the western samples of *E. yarraensis* , from the Otway Coast Basin (Aire and Gellibrand rivers), are highly divergent and show patterns inconsistent with localised geographic divergence (Figure S3B) at the level observed within the other species. Geographic‐based patterns of divergence among the eastern samples of *E. yarraensis* are of a similar magnitude to that seen in *E. armatus* and *E. bispinosus* and indicate that the easterly populations from the Yarra River (MON) and Werribee River (WER) are somewhat divergent from the three samples from the mid‐range of the species (BIR, LET, ELI), all from the adjacent Moorabool and Barwon river basins (Figures 1, 4, and S3B).

### 3.3 Utility of Mitochondrial and Ribosomal Gene Sequences

Comparisons of the average intra‐ and inter‐ specific variation among the target species and *E. spinifer* , and the length of the five ribosomal genetic elements (18S, 5.8S, ITS2, 28S and ETS) and 15 mitochondrial genes (13 mitochondrial protein‐coding genes and 2 ribosomal genes) used for the phylogenetic analyses are summarised in Figure S4. Average intra‐ and inter‐ specific values show limited overlap thereby giving most gene regions a degree of utility at the taxonomic levels under. One exception is the 5.8S gene which displays only 2 bp differences (0.62% divergence) between the outgroup species, *E. spinifer* and the 3 ingroup species. The other ribosomal markers show a greater range in levels of variability from the highly conserved 18S gene to the ETS region that shows divergence levels ranging over 50%. The ITS2 genetic marker is also quite variable with inter‐specific divergence levels in the order of 20%. The different ribosomal elements show significant length variation ranging from the short 5.8S (162 bp) gene to the 28S gene (approximately 5000 bp). While the sequences were not used in the analyses for this study, the ITS1 region showed extreme levels of length variation. The majority of this was inter‐specific, with this region ranging in length from 3998 to 4031 bp in *E. armatus* , through 2919 to 2925 in the *E. yarraensis* western lineage, 2497 to 2514 in the *E. yarraensis* eastern lineage, from 413 to 426 in *E. bispinosus* , and from 405 to 407 bp in *E. spinifer* . As a result, the alignment of these sequences resulted in major gaps and relatively low phylogenetic information content.

In comparison, the mitochondrial genetic elements examined showed a much‐reduced range of variability in divergence levels and lengths. The mitogenome in its entirety showed a level of divergence broadly similar to the 28S gene. The two mitochondrial ribosomal genes (16S and 12S) are the most conserved and contrast with the CR sequences which showed significantly elevated variability (up to 20%). The COI gene shows similar levels of divergence compared to the average for the mitochondrial genes, and is not greatly different from the other mitochondrial protein‐coding genes, but is significantly more divergent than the 16S and 12S genes.

Summaries of the relationships among samples using UPGMA clustering based on the individual ribosomal genetic elements and the pooled data are shown in Figure S5. With the exception of the 5.8S gene, each provides useful information with the four *Euastacus* species being identified, and all but the ITS2 region highlighting the geographic differentiation in *E. yarraensis* . A feature of the ribosomal data is the presence of both minor and major gaps in the alignment (Figure S6). An analysis of the complete ribosomal data using the UPGMA method, with and without the pairwise deletion of gaps (Figure S7), indicates that several groups show elevated levels of divergence with the inclusion of gaps, including the eastern and western *E. yarraensis* lineages and the geographically isolated *E. armatus* northern populations (KEL and BAL).

A comparison between the relationships determined by the commonly used barcoding gene regions for freshwater crayfish, COI (669 bp alignment) and 16S (531 bp alignment) together with the complete CR (1335 bp alignment) and the complete mitogenome (16,415 bp alignment) are shown in Figure S8. Superimposed on the dendrograms are the results of the taxonomic discrimination pipelines. The COI results are consistent with the earlier analyses (Figure 4), with 4 species identified by both the bPTP and ASAP methods and with average inter‐specific divergence levels ranging from 2.38% to 5.52%. The 16S‐based relationships and associated analyses fail to identify 2 taxonomic units within *E. yarraensis* and show low levels of inter‐specific divergence (0.76% to 1.78%). The CR and complete mitogenome sequences are largely congruent and the pipelines largely recover the same groupings. An exception is that the bPTP pipeline recovers some anomalous taxonomic units among the eastern *E. yarraensis* samples, and in the case of the CR data, one divergent sample from *E. bispinosus* (GRA) was identified as a putative species.

## 4 Discussion

Conservation biologists have been quick to adopt new genetic technologies and procedures that can support the understanding and protecting of intra and inter‐specific biodiversity. Genome skimming is one such relatively new genetic technology that is now increasingly popular, but most applications have been applied to phylogenetics or evolutionary questions and have generally used limited taxon sampling and focused mostly on the recovery of mitochondrial genomes in animal‐based studies (Gan et al. 2018; Machado et al. 2016; Taite et al. 2023; Tan et al. 2018; Trevisan et al. 2019; Wang et al. 2025). More recently, the 18S and 28S genes from the nuclear ribosomal cluster are being routinely extracted and sometimes also histone genes (Chang et al. 2024; Grandjean et al. 2017; Hoban et al. 2022). Our study is a novel example of genome skimming being used to recover both mitogenomes and all elements of the ribosomal complex (18S, ITS1, ITS2, 28S and partial ETS) focusing on the distribution of genetic variation within and between three closely related species within a conservation setting.

### 4.1 Taxonomic Boundaries

The existing taxonomic status and relationship for *E. armatus* and *E. bispinosus* were supported by gap analysis, using both the bPTP and ASAP method, on a larger sampling of genes and populations than has previously been undertaken (Avery and Austin 1997; Shull et al. 2005). The levels of inter‐ and intra‐specific variation in the mitochondrial and ribosomal genes in our study are broadly similar to the only other study of this kind on crayfish (Hammer et al. 2024). However, we did note higher rates of evolution in some ribosomal elements in *Euastacus* (see below), and the COI and 16S barcoding fragments have reduced levels of divergence compared with species of *Cherax* (Munasinghe et al. 2003; Nguyen et al. 2005).

Whilst the easterly samples of *E. yarraensis* formed a discrete lineage referable *sensu stricto* to *E. yarraensis* (Morgan 1986), we provide evidence for westerly samples from the Otway Ranges River Basin being highly divergent from all others analysed for the taxon. These samples group strongly with the *E. bispinosus* lineage, albeit in a basal position in the analysis of the mitogenome data set. Although based on more limited gene and population sampling, this pattern is also apparent in the phylogenetic trees presented in Shull et al. (2005), although this was not an aspect that these authors emphasised. The origin of the divergent mitogenomes in western *E. yarraensis*, while consistent with incipient speciation, could also be due to the retention of an ancestral mitochondrial haplotype or historical hybridisation (Thielsch et al. 2017). Incomplete lineage sorting of divergent mitochondrial haplotypes can lead to an overestimation of species or population divergence, misleading estimates of phylogenetic relationships and discordance with nuclear markers.

Relevant in this context is the finding that the ribosomal‐based phylogenetic reconstructions in the current study are discordant with the mitochondrial data in supporting *E. yarraensis* as a single monophyletic clade. Although it is noted that genetic distances between the western and easterly samples of this species were greater than those observed among samples within the *E. armatus* and *E. bispinosus* clades. Finding mitochondrial and nuclear gene discordance is unusual in crayfish (Grandjean et al. 2017; Hammer et al. 2024; Tan et al. 2021) but can be relatively common in some groups (Eriksen et al. 2025; Kimball et al. 2021; Perea et al. 2016) and complicates the determination of species boundaries. Nevertheless, the analyses point to substantial lineage diversification and isolation of western and eastern populations within *E. yarraensis* , which warrants further genetic analyses such as a SNP based approach and taxonomic review.

### 4.2 Intra‐Specific Variation and Phylogeography

Our study supports and improves understanding of the intra‐specific genetics across the *E. armatus* complex. The distinct and widespread lineage of *E. armatus* across the lower reaches of the Murray‐Murrumbidgee rivers matches the single panmictic population identified from nuclear microsatellite data by Whiterod et al. (2017). Our findings also support those of Whiterod et al. (2017) in *E. armatus* from Goodradigbee River–Talbingo Dam being divergent from all other locations, and the species from the upper Murray River (Towong and Ponderosa) also being divergent from all other samples from the Murray and Goodradigbee rivers. We further extend this knowledge by highlighting two additional phylogeographically divergent populations of *E. armatus* , one in mid to upper Ovens River and its tributaries and the second in the Goulburn River and Gunbower section of the mid‐Murray River. Collectively, these headwater populations contribute significantly to genetic variability within *E. armatus* .

A ‘prickly’ problem within *E. armatus* is the status of the two outlying northern populations, well removed from the Murray and Murrumbidgee river basins, in the upper reaches of the Lachlan and Macquarie‐Bogan river basins (BAL1‐4, KEL1‐4). Whiterod et al. (2017) found these populations to be genetically divergent from each other and all other sampled populations of *E. armatus* . A figure summarising the relationships among samples derived from their study based on pairwise Fst values is provided (Figure S9), which demonstrates that samples from these 2 northern locations are extreme outliers. However, due to anecdotal evidence of translocations of *E. armatus* to this river basin, Whiterod et al. (2017) attributed the divergence in microsatellite markers to be the likely result of drift due to small founder population size. In contrast, our findings show that each of these two populations is divergent, indicating these could be remnant populations from when the species may have had a much larger distributional range that encompassed the Lachlan and Darling River basins during cooler and wetter climatic periods, rather than the product of human‐mediated translocation. As a result, these northern populations also contribute significantly to the genetic diversity within *E. armatus* and therefore expand their natural distribution and conservation management requirements. A SNP‐based approach to assessing population variability and inter‐population relationships would be an obvious next step, as has been applied to other crayfish species (Unmack, Young, et al. 2019).

The levels of intra‐specific variation within *E. bispinosus* (and *E. yarraensis* —excluding the western clade samples) are of a similar order of magnitude to that observed in *E. armatus* considering their more restricted distributions. In *E. bispinosus* , the patterns of divergence are largely consistent with isolation by distance except for a single sample from Scrubby Creek (GRA) being unusually divergent. This observation is also consistent with a microsatellite study (Miller et al. 2014) which found this population to be divergent despite its close proximity to other populations within the Grampians National Park. The outlying population of *E. bispinosus* in the karst rising‐springs of the Millicent Coast Basin (BUB, EWE) can be considered a historically isolated population, given the degree of genetic divergence, rather than being the result of recent translocations. In contrast, the clustering of the outlying Fitzroy River population sample (TYR) with those from the Grampians National Park in the upper Glenelg River Basin supports the notion of this population arising from translocation, which is consistent with anecdotal reports and conclusions previously drawn from microsatellite studies (Miller et al. 2013, 2014).

### 4.3 Genome Skimming and the Use of New Genetic Markers

Most genome skimming studies that utilise mitogenome and nuclear gene information have focussed on technique development and phylogenetic questions with generally limited sampling and at high taxonomic levels (Grandjean et al. 2017; Taite et al. 2023; Wang et al. 2025). Our results demonstrate that data generated from genome skimming can be used to successfully address questions relevant to taxonomy and conservation at, and below, the species level. This study, and Hammer et al. (2024), demonstrate that additional regions can be extracted and utilised from the ribosomal gene cluster including the ITS1, ITS2, 5.8S and ETS sequences. The non‐transcribed ribosomal elements (ITS1, ITS2, ETS) show greater variation in length and nucleotide variability than the transcribed genes (18S, 5.8S, 28S) and the mitochondrial genes and control region, and these patterns are consistent with observations from other species (Lunerová et al. 2017; Stage and Eickbush 2007). The ETS region was replete with indels, giving higher levels of diversity, but almost all the major indels were distributed among species, thereby providing potentially useful taxonomic information. The most conserved gene was the 5.8S, which only displayed variability between the ingroup and outgroup, is not often used for systematic studies due to its short length. The 18S gene was relatively conserved compared to the other ribosomal elements, consistent with its frequent use for phylogenetic genetic studies at higher taxonomic levels (Bracken‐Grissom et al. 2014; Tsagkogeorga et al. 2009; Xia et al. 2003). However, this gene also displayed systematically useful information at the species level with several nucleotide sites allowing consistent discrimination of each of the four species of *Euastacus* in this study, which is unusual for crayfish (Grandjean et al. 2017; Hammer et al. 2024).

### 4.4 Conservation Implications

The study reveals the existence of significant phylogeographic structure within *E. armatus* , consisting of up to seven distinct lineages, some of which may correspond to Conservation Units or even Evolutionarily Sustaining Conservation Units as defined by Hoelzel (2023). Apart from one wide‐ranging lineage, these represent populations almost entirely associated with headwaters of major river basins. The two northern lineages, which may represent remnant populations rather than the result of translocations, are known to have reduced genetic variability and to be divergent on the basis of neutral genetic markers (Whiterod et al. 2017), and therefore need to be incorporated into the conservation management framework for the species. Of these two lineages, the population from the Cudgegong River now adds, along with the Critically Endangered Cudgegong giant spiny crayfish *Euastacus vesper* (McCormack and Ahyong 2017), to the conservation importance of the upper reaches of this river basin. The phylogeographic insight generated for *E. armatus* will be useful for guiding targeted conservation strategies, including all aspects of conservation translocation, which are now becoming increasingly necessary for the species (Whiterod et al. 2021).

Confirmation that populations of *E. bispinosus* in the karst rising‐springs are genetically distinct highlights the urgency for immediate genetic rescue due to critically low genetic diversity revealed by nuclear markers (Hoffmann et al. 2021; Miller et al. 2014). Whilst the translocated Portland Coast Basin *E. bispinosus* population can be considered as being of less importance genetically, this refuge population, outside the natural range of the species, may play a conservation role for an otherwise range‐restricted and threatened taxon into the future.

The finding of deep divergence within *E. yarraensis* suggests two taxa, one a formally described species and the other a formerly unknown candidate species (sensu Vences et al. 2005). This necessitates detailed morphological analysis, combined with the molecular data, in an integrative taxonomic review to establish diagnosability of the two taxa. If successful and the candidate taxon is formally described, this will prompt elevation of conservation status of both species and afford the ability to protect this diversity through legislation and other formal mechanisms (Ely et al. 2017).

## 5 Conclusions

The genus *Euastacus* is among the most threatened crayfish genera in the world (Richman et al. 2015) with concerns of the loss of phylogenetic diversity under future climates (Pipins et al. 2025). The present study of an important *Euastacus* complex has demonstrated that genome skimming to generate whole mitogenomes and nuclear markers has the potential to reveal valuable taxonomic and phylogeographic knowledge. Extending this research comprehensively across the *Euastacus* will enhance similar insight to guide the prioritisation of conservation actions that can lessen extinction risk across members in the genus.

## Author Contributions

**Christopher M. Austin:** conceptualisation (equal), data curation (equal), funding acquisition (equal), investigation (equal), methodology (equal), project administration (equal), writing – original draft (equal), writing – review and editing (equal). **Shane T. Ahyong:** investigation (equal), resources (equal), writing – review and editing (equal). **Adam Miller:** investigation (equal), resources (equal), writing – review and editing (equal). **Tarmo A. Raadik:** investigation (equal), resources (equal), writing – review and editing (equal). **Mark Lintermans:** investigation (equal), resources (equal), writing – review and editing (equal). **Rob McCormack:** investigation (equal), resources (equal), writing – review and editing (equal). **Sylvia Zukowski:** investigation (equal), writing – review and editing (equal). **Michael P. Hammer:** investigation (equal), writing – review and editing (equal). **Frederic Grandjean:** investigation (equal), writing – review and editing (equal). **Dean M. Gilligan:** investigation (equal), writing – review and editing (equal). **Matt Beitzel:** investigation (equal), writing – review and editing (equal). **Nick S. Whiterod:** conceptualisation (equal), funding acquisition (equal), investigation (equal), methodology (equal), project administration (equal), resources (equal), writing – original draft (equal), writing – review and editing (equal).

## Funding

This work was partially supported by the Threatened Species Initiative and the Australian Government's Bushfire Recovery for Wildlife and their Habitats.

## Conflicts of Interest

The authors declare no conflicts of interest.

## Supporting information


**Table S1:** Summary of the site locations and sample sources utilised in the present study.
**Table S2:** Assembly statistics for all samples sourced through genome skimming in the current study, sample code location data provided in Table S1 (SRA = NCBI's Sequence Read Archive, TSI = Threatened Species Initiative, Total reads = trimmed values, RL = sequence read length, contig lengths given in base pairs, Accession = GenBank).
**Table S3:** Diversity statistics derived form the mitogenome data set for *E. armatus* samples and geographically‐based subgroups identified from phyogenetic and network analyses.

**Figure S1:** Summary of PCR‐based sequences available on NCBI at the commencement of this study for *E. armatus* , *E. bispinosus* and *E. yarraensis* .

**Figure S2:** Multidimensional scaling (MDS) analysis based on mitogenome distances between samples using the JC69 model. (A) all samples, (B) *E. armatus* samples. See Figure 1 for geographic locations and colour coding of samples.

**Figure S3:** Minimum Spanning Network analyses for *E. bispinosus* (A) and *E. yarraensis* (B) based on full mitogenome sequences. Ticks on branches connecting samples represent mutational steps.

**Figure S4:** Length of genes and genetic elements used for phylogenetic and distance‐based analyses and the ITS1 region (A); divergence levels within and between species for each gene and genetic element based KC69 distance (B).

**Figure S5:** Comparison of UPGMA summaries of genetic relationships among species of *Euastacus* using the KC69 model for each ribosomal element and the combined ribosomal data (ALL). Average divergence between the outgroup and in group samples is given as a percentage. Colour coding for OTUs follows Figure 2.

**Figure S6:** Visual representation of alignment of ribosomal contigs used in this study with genetic elements and species highlighted for 57 *Euastacus* samples (sample MON deleted).

**Figure S7:** Comparison of UPGMA summaries using p‐distances with alignment gaps included (A), and with pairwise deletion (B) using the P*R*O*P web portal. Key elements differing between the analyses are emphasised with arrows. Green arrow applies to *E. bispinosus* samples; pink and yellow arrows highlight the divergence levels in *E. yarraensis* and the purple and blue arrows highlight divergence levels in the isolated northern *E. armatus* populations (KEL and BAL).

**Figure S8:** Comparison of the results of barcoding gap analysis using the ASAP and bPTP web‐portals, based on the COI barcoding gene fragment (669 bp alignment), the 16S barcoding (531 bp alignment), the control region (1335 bp alignment) and the complete mitogenome (16,415 bp alignment). Colour coding for OTUs follows Figure 2, with coloured bar and/or offset bars representing putative species as identified by the ASAP and bPTP pipelines. Divergence as % is given at major nodes.

**Figure S9:** UPGMA summary of pair‐wise Fst values from Whiterod et al. (2017). Location codes are those of Whiterod et al. (2017), with the corresponding codes used in this study in parentheses. * indicates where there is not a one to one correspondence for samples sites between studies.

## Data Availability

Raw sequences generated in this study are available from the NCBI Sequence Read Archive and the TSI Sequence Archive. Mitogenome, 18S and 28S contig nucleotide sequences for each sample are available from the NCBI's nucleotide archive using the accession numbers given in Table S2.
