# Supplementary figures and images for: A Prickly Problem: Genome Skimming Reveals Varying Levels of Phylogenetic Diversity in the Freshwater Crayfish Euastacus armatus Complex (Parastacidae) With Implications for Taxonomy and Conservation
Source: Ecol Evol. 2026 Apr 20;16(4):e73428. doi: 10.1002/ece3.73428 (PMC13096719; doi:10.1002/ece3.73428)

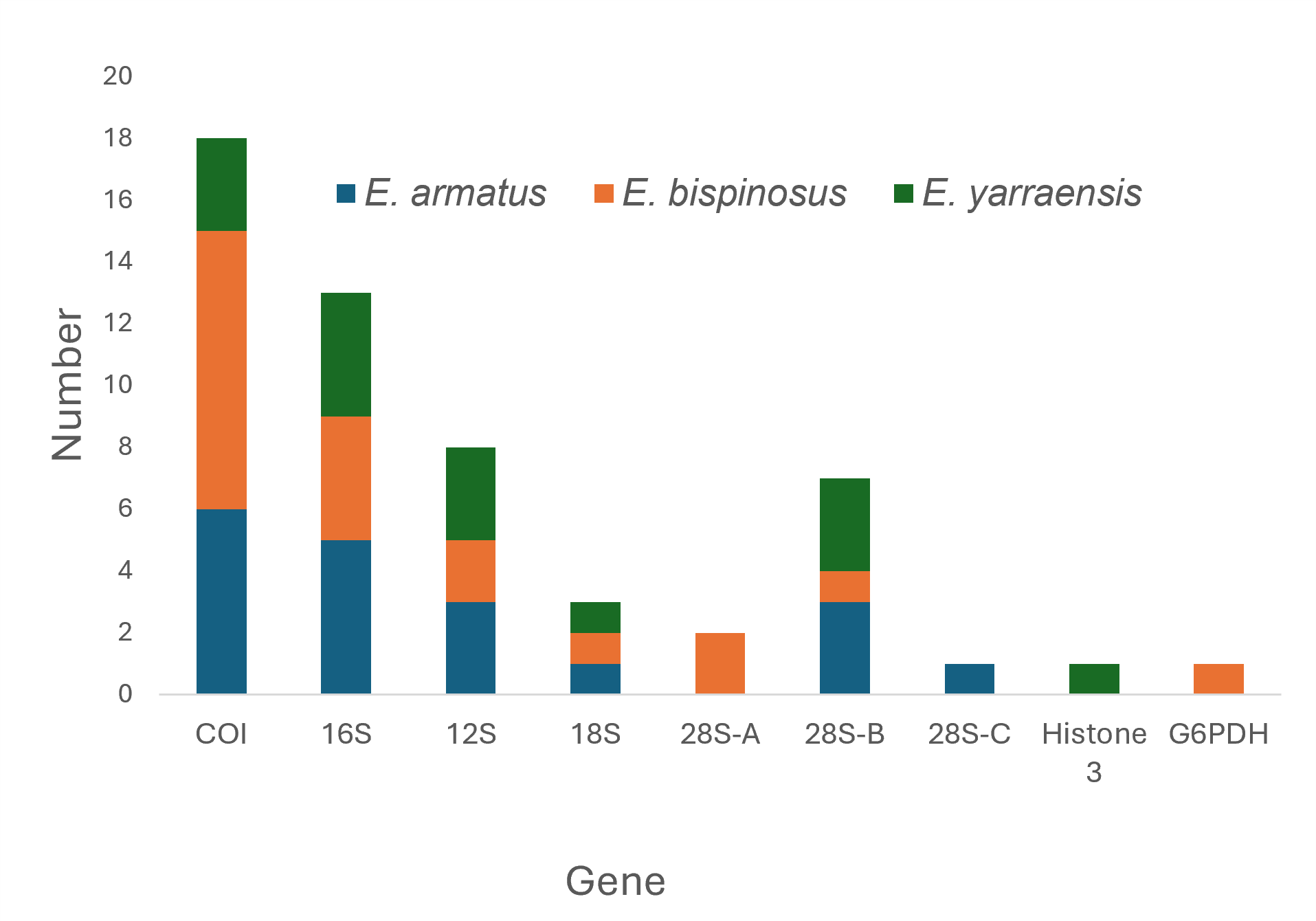

Supplement: Supplementary file 2 — Figure S1: Summary of PCR‐based sequences available on NCBI at the commencement of this study for E. armatus , E. bispinosus and E. yarraensis . [file ECE3-16-e73428-s006.tif]

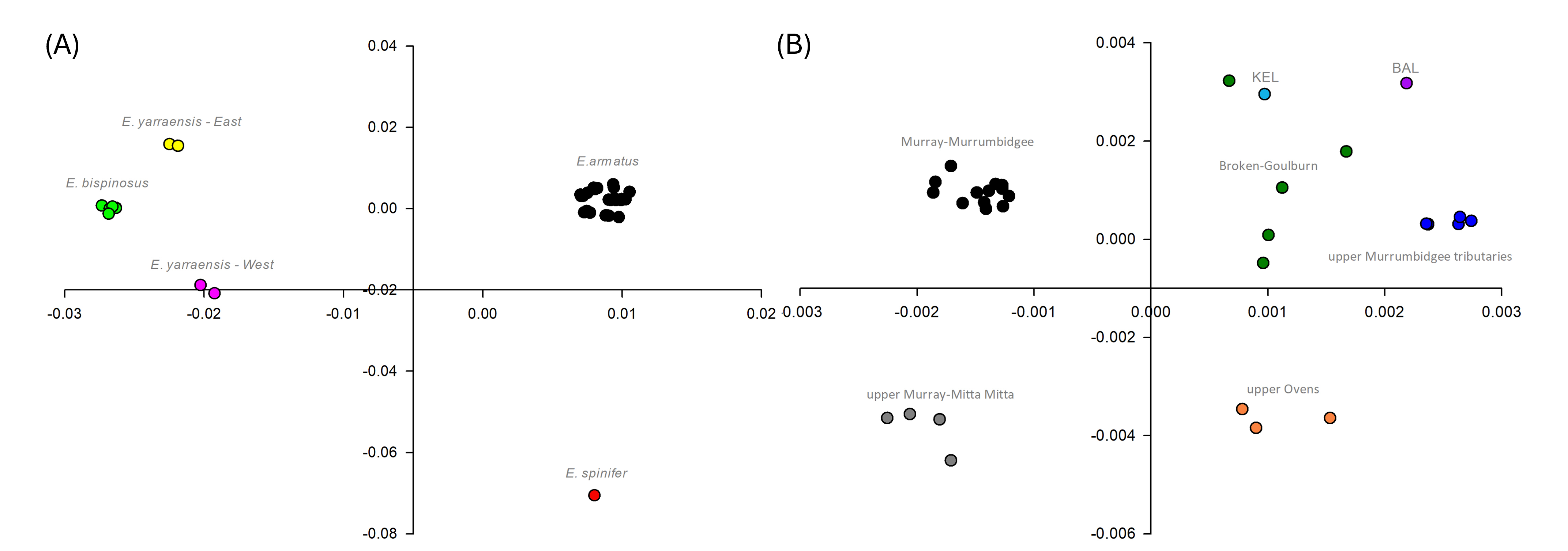

Supplement: Supplementary file 3 — Figure S2: Multidimensional scaling (MDS) analysis based on mitogenome distances between samples using the JC69 model. (A) all samples, (B) E. armatus samples. See Figure 1 for geographic locations and colour coding of samples. [file ECE3-16-e73428-s010.tif]

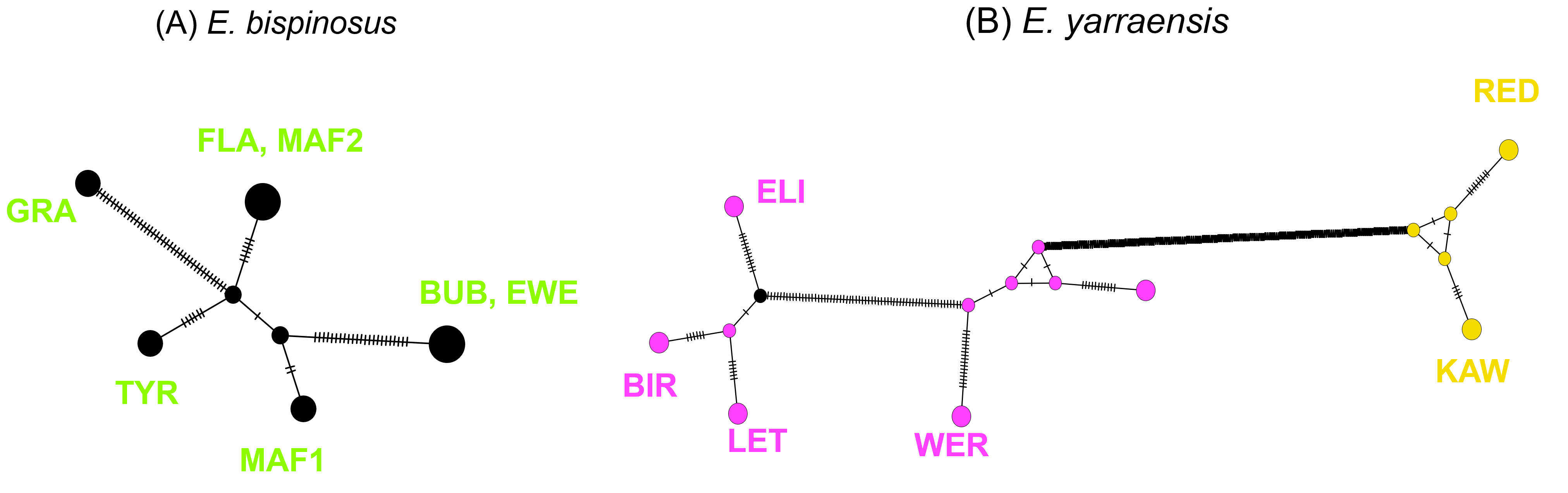

Supplement: Supplementary file 4 — Figure S3: Minimum Spanning Network analyses for E. bispinosus (A) and E. yarraensis (B) based on full mitogenome sequences. Ticks on branches connecting samples represent mutational steps. [file ECE3-16-e73428-s005.tif]

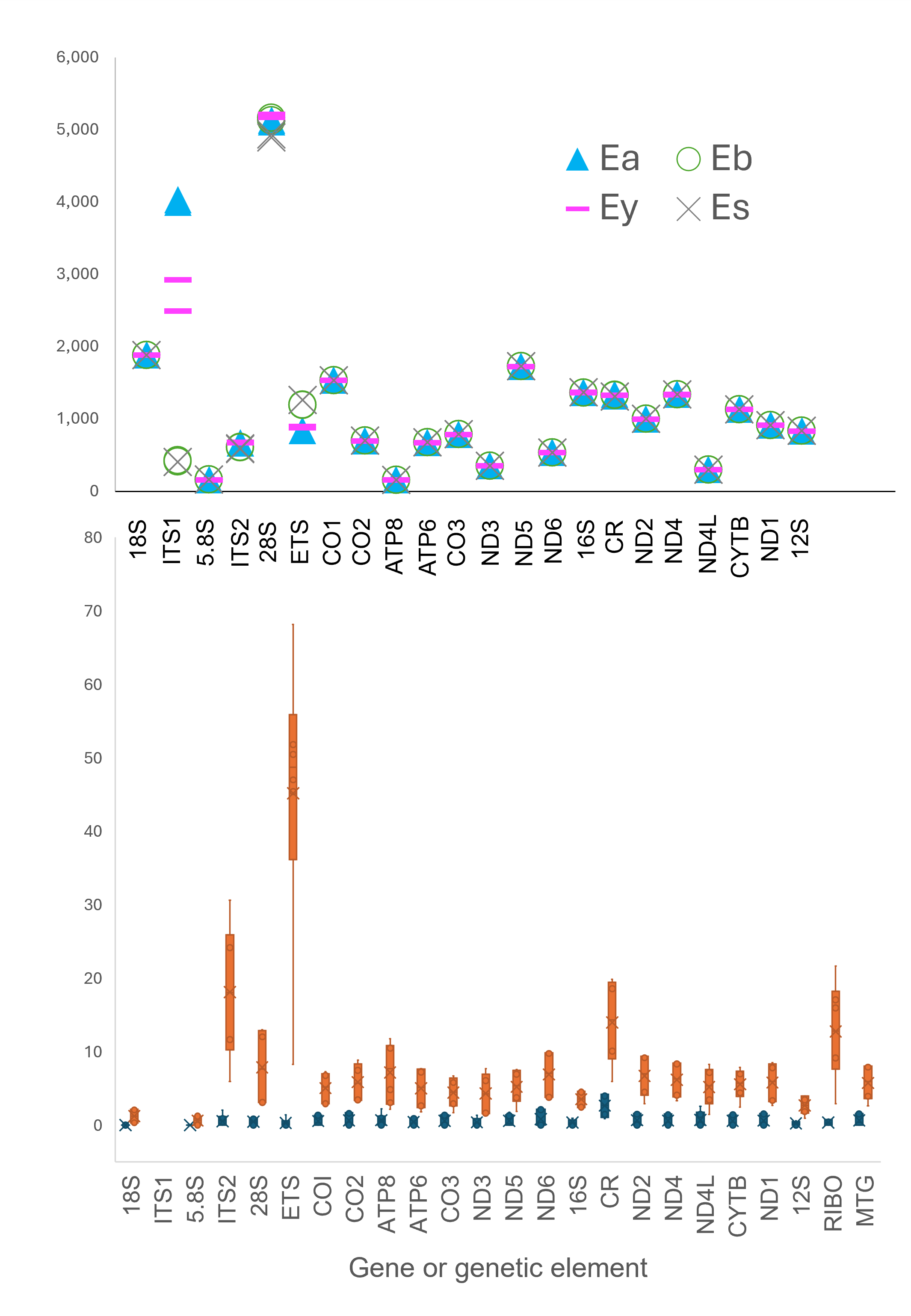

Supplement: Supplementary file 5 — Figure S4: Length of genes and genetic elements used for phylogenetic and distance‐based analyses and the ITS1 region (A); divergence levels within and between species for each gene and genetic element based KC69 distance (B). [file ECE3-16-e73428-s008.tif]

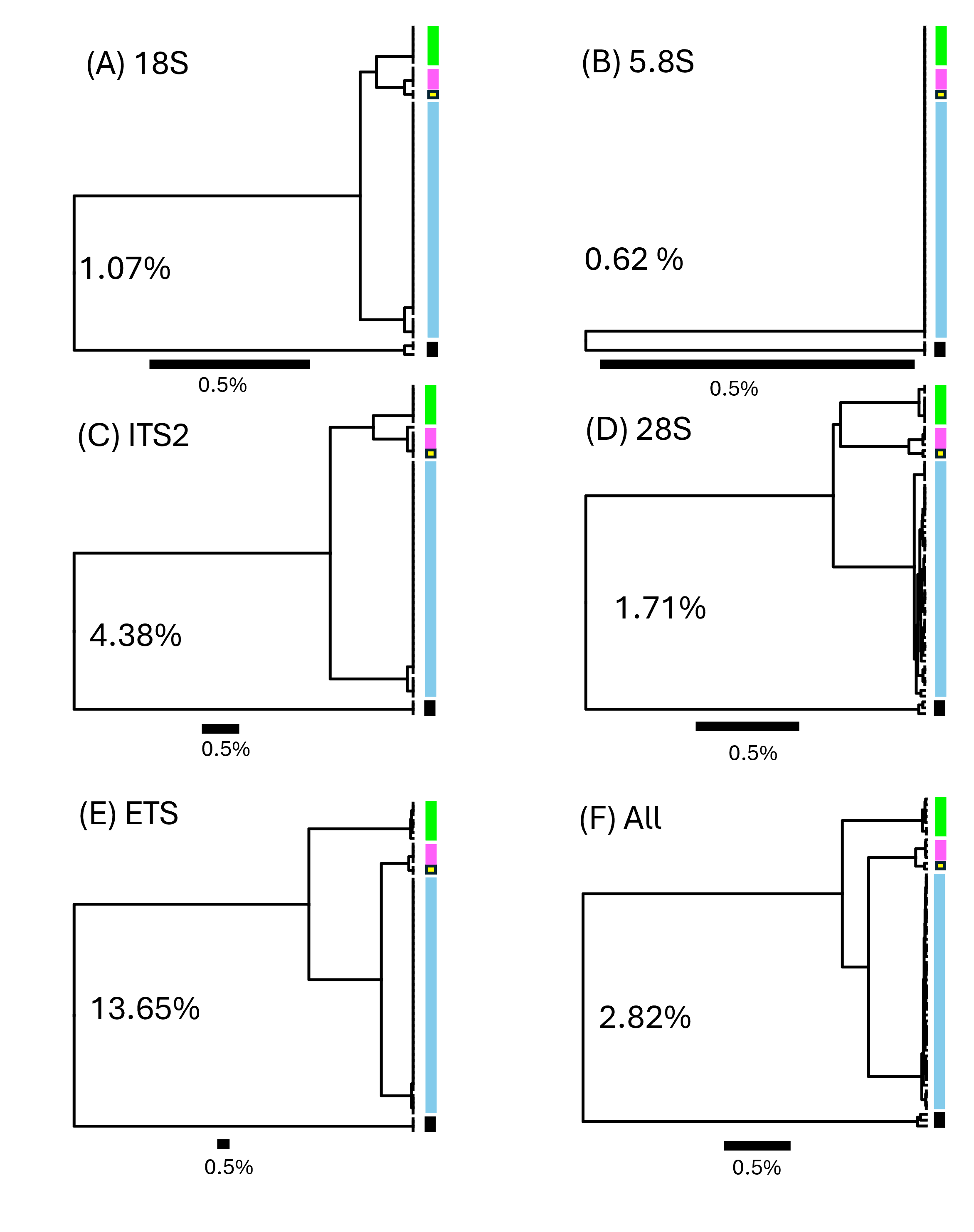

Supplement: Supplementary file 6 — Figure S5: Comparison of UPGMA summaries of genetic relationships among species of Euastacus using the KC69 model for each ribosomal element and the combined ribosomal data (ALL). Average divergence between the outgroup and in group samples is given as a percentage. Colour coding for OTUs follows Figure 2. [file ECE3-16-e73428-s009.tif]

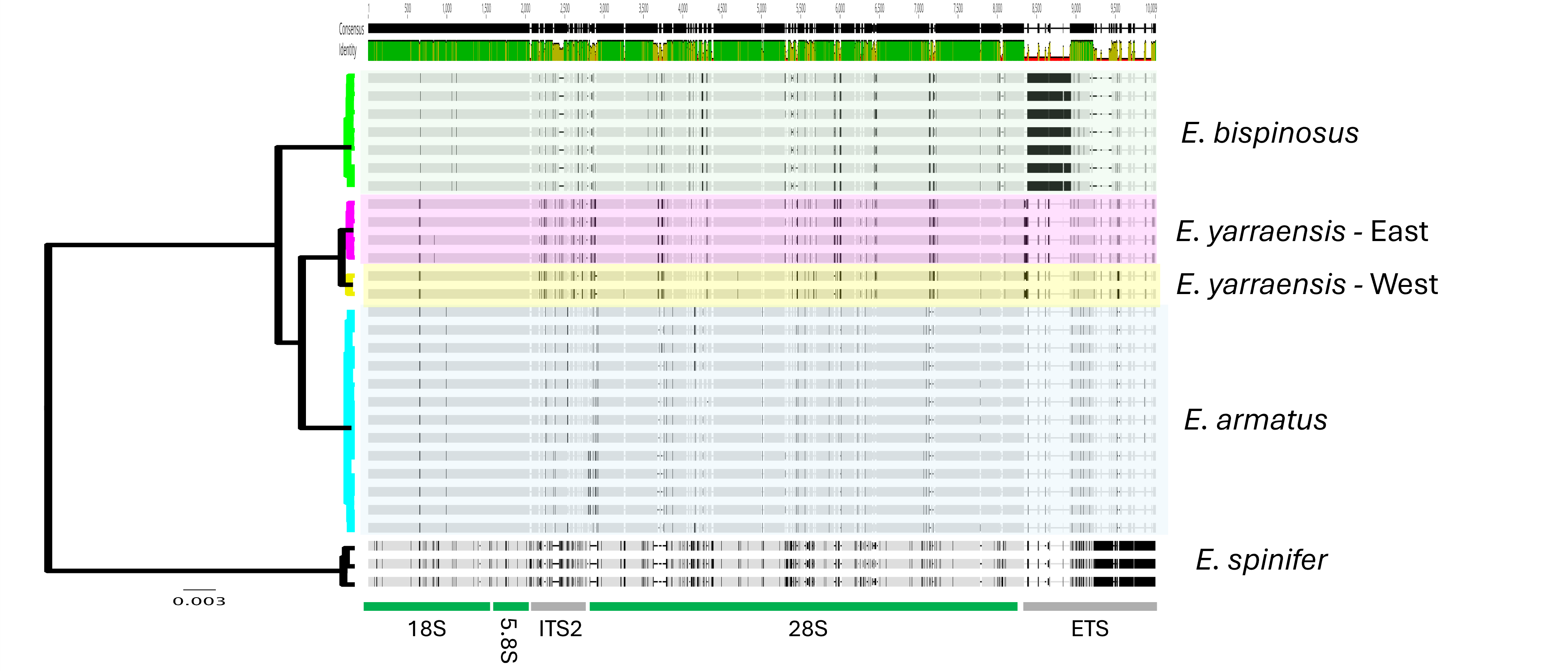

Supplement: Supplementary file 7 — Figure S6: Visual representation of alignment of ribosomal contigs used in this study with genetic elements and species highlighted for 57 Euastacus samples (sample MON deleted). [file ECE3-16-e73428-s007.tif]

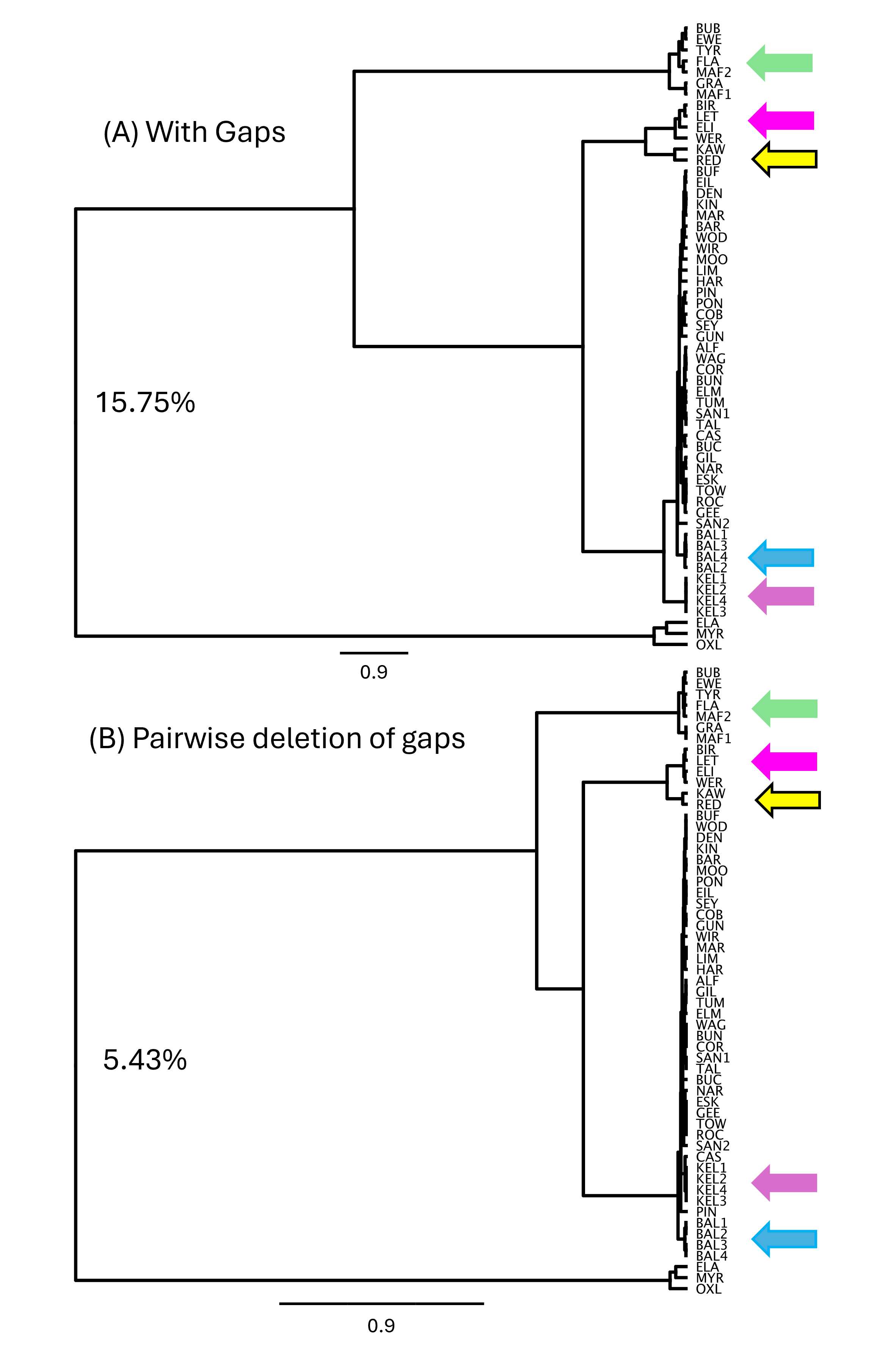

Supplement: Supplementary file 8 — Figure S7: Comparison of UPGMA summaries using p‐distances with alignment gaps included (A), and with pairwise deletion (B) using the P*R*O*P web portal. Key elements differing between the analyses are emphasised with arrows. Green arrow applies to E. bispinosus samples; pink and yellow arrows highlight the divergence levels in E. yarraensis and the purple and blue arrows highlight divergence levels in the isolated northern E. armatus populations (KEL and BAL). [file ECE3-16-e73428-s003.tif]

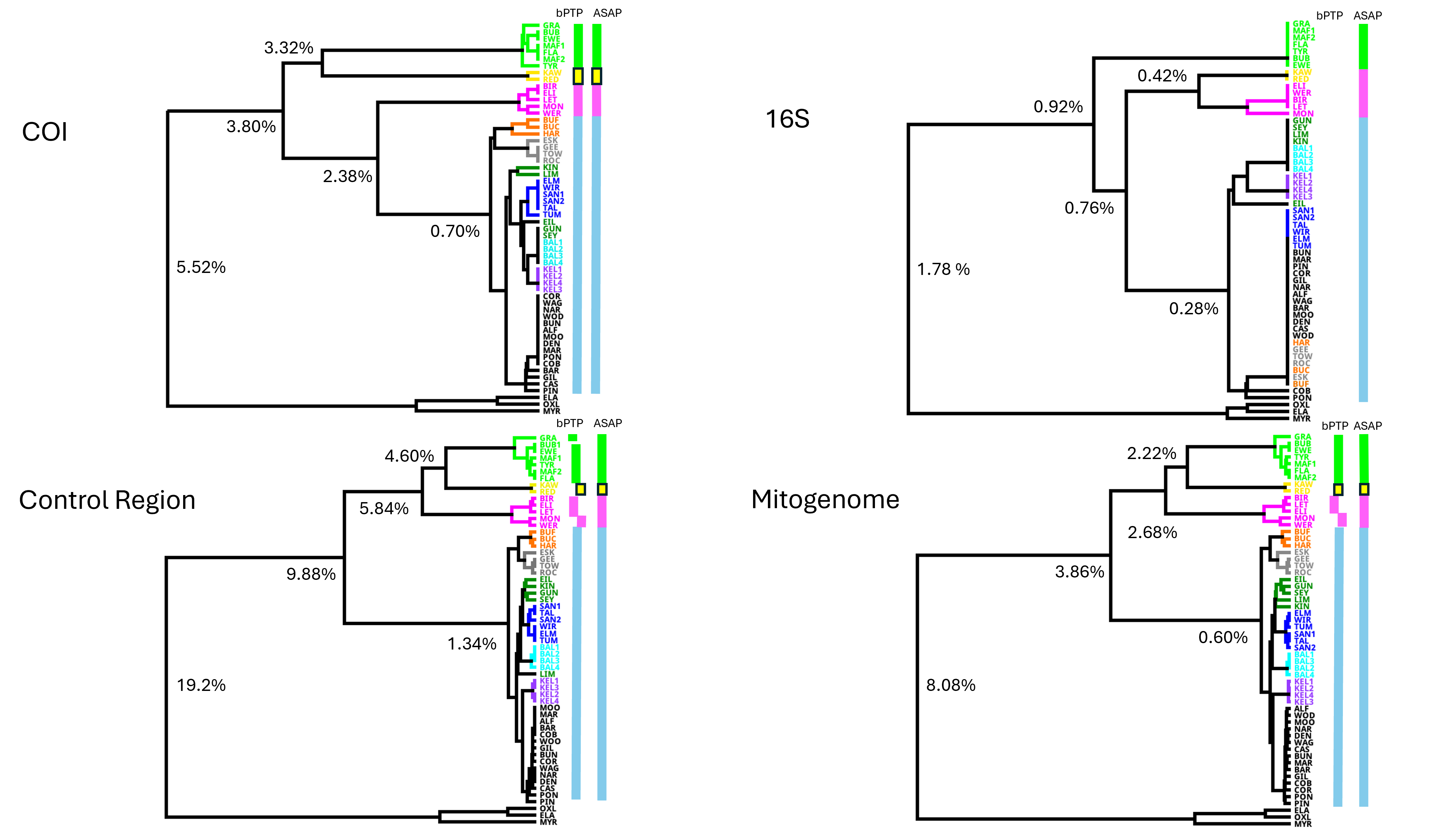

Supplement: Supplementary file 9 — Figure S8: Comparison of the results of barcoding gap analysis using the ASAP and bPTP web‐portals, based on the COI barcoding gene fragment (669 bp alignment), the 16S barcoding (531 bp alignment), the control region (1335 bp alignment) and the complete mitogenome (16,415 bp alignment). Colour coding for OTUs follows Figure 2, with coloured bar and/or offset bars representing putative species as identified by the ASAP and bPTP pipelines. Divergence as % is given at major nodes. [file ECE3-16-e73428-s002.tif]

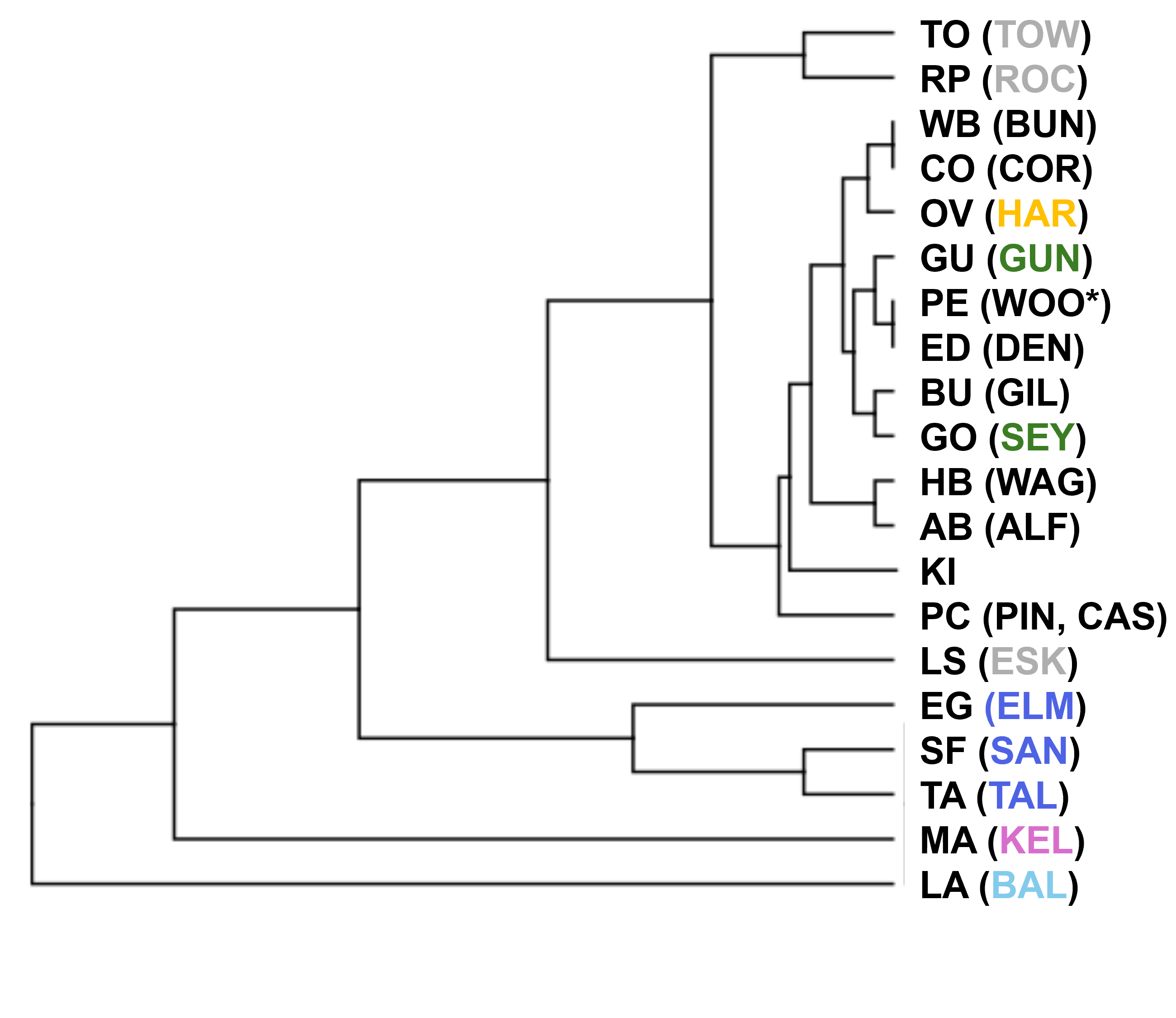

Supplement: Supplementary file 10 — Figure S9: UPGMA summary of pair‐wise Fst values from Whiterod et al. (2017). Location codes are those of Whiterod et al. (2017), with the corresponding codes used in this study in parentheses. * indicates where there is not a one to one correspondence for samples sites between studies. [file ECE3-16-e73428-s004.tif]
